# Adjusting soluble transferrin receptor concentrations for inflammation: Biomarkers Reflecting Inflammation and Nutritional Determinants of Anemia (BRINDA) project

**DOI:** 10.3945/ajcn.116.142232

**Published:** 2017-06-14

**Authors:** Fabian Rohner, Sorrel ML Namaste, Leila M Larson, O Yaw Addo, Zuguo Mei, Parminder S Suchdev, Anne M Williams, Fayrouz A Sakr Ashour, Rahul Rawat, Daniel J Raiten, Christine A Northrop-Clewes

**Affiliations:** 1 GroundWork, Fläsch, Switzerland;; 2 Strengthening Partnerships, Results, and Innovations in Nutrition Globally, Arlington, VA;; 3 Helen Keller International, Washington, DC;; 4 Emory University, Atlanta, GA;; 5 Nutrition Branch, CDC, Atlanta, GA;; 6 Department of Nutrition and Food Science, University of Maryland, College Park, MD;; 7 International Food Policy Research Institute, Washington, DC;; 8 *Eunice Kennedy Shriver* National Institute of Child Health and Human Development, NIH, Bethesda, MD; and; 9 Independent Public Health Nutrition Consultant, Cambridge, United Kingdom

**Keywords:** anemia, inflammation, iron deficiency, nutritional assessment, soluble transferrin receptor

## Abstract

**Background:** Iron deficiency is thought to be one of the most prevalent micronutrient deficiencies globally, but an accurate assessment in populations who are frequently exposed to infections is impeded by the inflammatory response, which causes iron-biomarker alterations.

**Objectives:** We assessed the relation between soluble transferrin receptor (sTfR) concentrations and inflammation and malaria in preschool children (PSC) (age range: 6–59 mo) and women of reproductive age (WRA) (age range: 15–49 y) and investigated adjustment algorithms to account for these effects.

**Design:** Cross-sectional data from the Biomarkers Reflecting the Inflammation and Nutritional Determinants of Anemia (BRINDA) project from 11,913 PSC in 11 surveys and from 11,173 WRA in 7 surveys were analyzed individually and combined with the use of a meta-analysis. The following 3 adjustment approaches were compared with estimated iron-deficient erythropoiesis (sTfR concentration >8.3 mg/L): *1*) the exclusion of individuals with C-reactive protein (CRP) concentrations >5 mg/L or α-1-acid glycoprotein (AGP) concentrations >1 g/L, *2*) the application of arithmetic correction factors, and *3*) the use of regression approaches.

**Results:** The prevalence of elevated sTfR concentrations incrementally decreased as CRP and AGP deciles decreased for PSC and WRA, but the effect was more pronounced for AGP than for CRP. Depending on the approach used to adjust for inflammation, the estimated prevalence of iron-deficient erythropoiesis decreased by 4.4–14.6 and 0.3–9.5 percentage points in PSC and WRA, respectively, compared with unadjusted values. The correction-factor approach yielded a more modest reduction in the estimated prevalence of iron-deficient erythropoiesis than did the regression approach. Mostly, adjustment for malaria in addition to AGP did not significantly change the estimated prevalence of iron-deficient erythropoiesis.

**Conclusions:** sTfR may be useful to assess iron-deficient erythropoiesis, but inflammation influences its interpretation, and adjustment of sTfR for inflammation and malaria should be considered. More research is warranted to evaluate the proposed approaches in different settings, but this study contributes to the evidence on how and when to adjust sTfR for inflammation and malaria.

## INTRODUCTION

The accurate estimation of the prevalence and severity of iron deficiency in a population is important for programming and intervention strategies to reduce iron deficiency and anemia. An expert consultation held by the WHO and CDC in 2004 concluded that ferritin concentrations are reflective of liver iron stores, which are responsive to iron interventions, and should, therefore, be used to determine iron deficiency in populations with low levels of inflammation (1). Unfortunately, ferritin concentrations are increased during inflammation because ferritin is a positive acute-phase protein (APP); therefore, the interpretation of ferritin concentrations as a measure of iron deficiency becomes difficult in areas of high exposure to infection (2, 3). Where exposure to inflammation is high, soluble transferrin receptor (sTfR) concentrations alone or sTfR:ferritin ratios are recommended as alternate biomarkers because they are thought to be less affected by inflammation (4, 5).

When there is insufficient iron for the synthesis of hemoglobin, there is an increased expression of sTfR on the erythroblasts in the bone marrow, which also increases the concentrations of sTfR in circulating blood (6). sTfR is mainly expressed by erythroid precursors and, as such, sTfR concentrations reflect erythropoietic activity and cellular iron status (7). sTfR concentrations are proportional to the cellular iron demand and, thus, reflect early functional iron deficiency (6, 8).

Several reports that have described changes in sTfR concentrations during inflammation have been published (9–12); the degree of change varied across studies, but a consistent pattern of increased sTfR concentrations was reported. In a study that assessed the usefulness of sTfR as a biomarker of iron deficiency compared with that of bone marrow staining as the gold standard, the authors concluded that sTfR, albeit not optimal, was still one of the better-performing iron-status biomarkers in high-inflammation settings (13); however, only C-reactive protein (CRP) was measured to capture inflammation. Where malaria is endemic, malaria-induced hemolysis may lead to increased erythropoiesis (9, 14, 15). However, malaria may also induce changes in sTfR concentrations independent of both the acute-phase response and body iron content, although the magnitude and direction of this relation are not clear (16).

Currently, the APPs most frequently measured to assess inflammation are CRP and α-1-acid glycoprotein (AGP). Although CRP has been established as a biomarker of inflammation since the 1930s, the sensitivity of the method to estimate inflammation has improved significantly in recent years (17). AGP has been used as a measure of longer-term exposure to inflammation, but there is some uncertainty as to the threshold that defines elevated AGP when interpreting iron status.

Several approaches to address the issue of inflammation in the interpretation of nutritional biomarkers have been proposed (18, 19). The approaches have been described in the methodology overview of this supplement, which is an open access publication (3). Thus far, no consensus has been reached, to our knowledge, on whether or how to adjust for the effect of inflammation on sTfR concentrations. This article examines different approaches with the use of large multicountry data sets. Because the exposure to inflammation varies not only by geography but also by population subgroups, the scope of this article is limited to preschool children (PSC) (age range: 6–59 mo) and women of reproductive age (WRA) (age range: 15–49 y).

In this article, the following 4 questions are addressed: *1*) Is there a need to measure biomarkers of inflammation when sTfR is used as a biomarker of iron deficiency? *2*) Is there a need to measure >1 inflammation biomarker (CRP and AGP) to adjust sTfR? *3*) Is an additional adjustment needed to correct sTfR concentrations in the presence of malaria? and *4*) How do the different approaches for correcting for inflammation and for calculating the prevalence of iron-deficient erythropoiesis compare with each other?

## METHODS

### Data sources used

We used data from the Biomarkers Reflecting Inflammation and Nutritional Determinants of Anemia (BRINDA) project (www.BRINDA-nutrition.org) (20). The BRINDA protocol was reviewed by the institutional review boards of the NIH and was deemed to be non–human subjects research because the work is based on a secondary data analysis. The methods for identifying data sets, inclusion and exclusion criteria, and data management for the BRINDA project have been described in the methodologic overview in this supplement, which is an open access publication (3).

The surveys were nationally or regionally representative, and the inclusion criteria were as follows: *1*) surveys were conducted after 2004, *2*) target groups included PSC or nonpregnant WRA, and *3*) surveys measured ≥1 biomarker of iron (ferritin or sTfR) or vitamin A status (retinol-binding protein or retinol) and ≥1 biomarker of inflammation (AGP or CRP). Surveys included in this analysis were those with measures of sTfR and inflammation (CRP or AGP). On the basis of these inclusion criteria, of the 16 PSC and 10 WRA BRINDA data sets, data from 11 PSC and 7 WRA surveys were applicable for analysis for this article. Malaria was measured in 5 PSC and 3 WRA data sets. Both CRP and AGP were measured in 9 of 11 PSC data sets (*n* = 11 surveys of CRP; *n* = 9 surveys of AGP) and 5 of 7 WRA data sets (*n* = 7 surveys of CRP; *n* = 5 surveys of AGP). In all surveys in which WRA data were collected, PSC data were also collected as part of the same survey.

### Laboratory analysis

Venous or capillary blood was collected from each respondent, and plasma or serum was obtained via centrifugation of the blood and stored at −20°C until analysis; 1 survey used dried blood spots. sTfR, AGP, and CRP concentrations were assessed with the use of a sandwich ELISA at the VitMin Laboratory in 9 of 11 PSC data sets and 5 of 7 WRA data sets (21), whereas in-country methods were used for Mexico [sTfR: Dade Behring N-Latex sTfR analyzer, Dade Behring BNA 1000 (Dade Behring); CRP: nephelometry, Behring Nephelometer 100 Analyzer)] and the United States (sTfR and CRP: immunoturbidimetry, Roche/Hitachi 912 clinical analyzer).

Current malaria was assessed with the use of microscopy [Kenya and Côte d’Ivoire (22)], current or recent malaria was assessed with the use of the Paracheck Pf rapid diagnostic test (Orchid Biomedical System) in Liberia, and plasma histidine-rich protein 2 (Cellabs Pty Ltd.) was assessed in Cameroon. A standardizing malaria diagnostic was not used because a sensitivity analysis showed a minimal impact (23). Additional information on laboratory methods is further described in the methodologic overview in this supplement (3).

### Case definitions

Iron-deficient erythropoiesis was defined as an sTfR concentration >8.3 mg/L in both PSC and WRA. Because no widely accepted threshold for sTfR exists, the laboratory’s base cutoff of >8.3 mg/L with the use of the Ramco method (TFC-94) was used for results that were obtained from the VitMin Laboratory (21). In Mexico and the United States, sTfR concentrations were multiplied by 1.6 to account for Ramco-Roche assay differences (24). Malaria was defined as either positive or negative. Inflammation was defined as a CRP concentration >5mg/L or AGP concentration >1 g/L (2, 21). A household socioeconomic status asset score was defined by survey investigators who applied a principal component analysis within each survey to household characteristics and item ownership and compared the poorest wealth quintile to the higher wealth quintile. Maternal education was defined as any school compared with no school.

### Statistical analysis

Descriptive statistics were calculated with the use of STATA 12.0 software (StataCorp) and cross-checked with SAS 9.4 software (SAS Institute). Correlations between sTfR, CRP, AGP, and malaria were calculated with the use of Kendall’s τ correlation coefficients with the SOMERSD package (25). The Taylor linearization method was used to obtain unbiased estimates that incorporated the weight, strata, and cluster (as applicable) when analyzing individual surveys. To combine data, individual survey analyses, which accounted for the complex survey design, were done with the use of the “survey” package in R 3.2.2 software (R Core Team) (26). Then, the individual survey estimates were combined with the use of the meta-analysis approach with the R package “metafor” (27). The heterogeneity of estimates across the surveys was assessed with the use of Cochran’s heterogeneity test (25).

Several approaches to adjust sTfR for inflammation and malaria were explored (3). First, the prevalence of iron-deficient erythropoiesis (sTfR concentration >8.3 mg/L) was calculated before applying any adjustments to sTfR, which was referred to as the unadjusted estimate. Subsequently, 3 adjustment approaches were applied to account for inflammation and malaria as follows: *1*) the exclusion of subjects with any inflammation, *2*) correction factors (CFs), and *3*) regression corrections (RCs).

### Exclusion approach

The exclusion approach used the inflammation biomarker information to exclude individuals with elevated CRP or AGP (or both) from the analysis; this method resulted in a smaller sample size. In this work, we excluded individuals with AGP concentrations >1 g/L and calculated the estimated prevalence of iron-deficient erythropoiesis in the remaining individuals.

### CF approach

The CF approach, as proposed by Thurnham et al. (2), uses arithmetic CFs. We calculated CFs by grouping inflammation into 4 groups as follows: *1*) reference (both CRP concentration ≤5 mg/L and AGP concentration ≤1 g/L); *2*) incubation (CRP concentration >5 mg/L and AGP concentration ≤1 g/L); *3*) early convalescence (both CRP concentration >5 mg/L and AGP concentration >1 g/L); and *4*) late convalescence (CRP concentration ≤5 mg/L and AGP concentration >1 g/L). We also calculated CFs by grouping inflammation into 2 groups in which CRP and AGP were used independently. CFs were defined as the ratio of geometric means of the reference group (nonelevated CRP or AGP) to those of the respective inflammation groups. CFs were calculated with the use of individual survey-specific data [termed here internal correction factor (ICF)] and from BRINDA’s meta-analysis values [termed the BRINDA correction factor (BCF)]. Although we initially ran the CFs for both AGP and CRP, we decided to not include CRP in the final models because of the underlying physiologic mechanism wherein elevated CRP prevents the rise of sTfR during the early acute-phase response and because the adjustments were minimal and somewhat inconsistent (see Results).

Similar adjustments to sTfR on the basis of malaria were also explored in populations in whom malaria is endemic. sTfR was adjusted with the use of an ICF to account for malaria (2-group CF: malaria negative compared with malaria positive) as well as ICFs to account for both long-term inflammation and malaria (4 group CFs: malaria negative and AGP concentration ≤1 g/L, malaria negative and AGP concentration >1 g/L, malaria positive and AGP concentration ≤1 g/L, and malaria positive and AGP concentration >1 g/L).

### RC approach

The RC approach uses linear regression to adjust sTfR by the concentration of AGP on a continuous scale and with malaria as a dichotomous variable (3). In brief, the adjusted sTfR equation was calculated by subtracting the influence of AGP and malaria as follows:


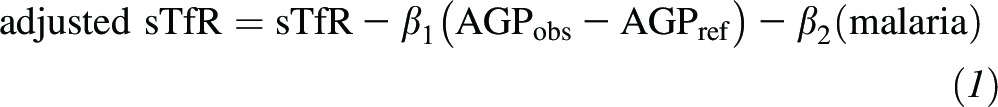


Depending on the available data, AGP or malaria can be included in the model. β_1_ is the AGP regression coefficient, β_2_ is the malaria regression coefficient, obs is the observed value, and ref is the reference value generated to define low inflammation (maximum value of the lowest AGP decile with the use of combined BRINDA data with non-logged reference values as follows: AGP in PSC: 0.59; AGP in WRA: 0.54). AGP and sTfR are all ln transformed; AGP is a continuous variable, and malaria is a dichotomous variable. The correction was only applied to individuals with ln AGP greater than ln AGP_ref_ to avoid overadjustments (3). An illustrative example of the use of the RC approach to adjust sTfR for AGP and CRP in PSC in Liberia is provided in **Supplemental Figure 1**.

The first step in the RC approach was to ln transform sTfR and AGP concentrations to approximate normality on the basis of regression diagnostics. Second, linear regression coefficients for AGP or malaria were obtained (bivariate and multivariate) with sTfr as the outcome. A test of multicollinearity between malaria and ln AGP was assessed on the basis of a test of tolerance (>0.1) and variance inflation factor (<5) to determine whether it was appropriate to include all variables in the model. Third, an ln-AGP reference value was subtracted from ln-AGP concentrations in the regression equation.

The RC approach is presented based on each individual survey (internal regression correction (IRC) and with the use of slope estimates from a BRINDA meta-analysis [termed BRINDA regression corrections (BRCs)]). The BRC approach entailed replacing the AGP β coefficients in the adjusted sTfR Equation *1* with the meta-analysis β coefficients. The same external reference value was used when applying both the IRC and BRC approaches. The IRC approach was performed for AGP alone or for both AGP and malaria. The BRC approach was not applied when including malaria in the model because meta-analysis β coefficients were not generated in this subpopulation because of the limited number of surveys that measured malaria.

### Comparison of adjustments

Unadjusted and adjusted prevalence estimates of iron-deficient erythropoiesis were compared with the use of McNemar’s chi-square test; statistical significance was defined as *P* < 0.05 before applying Bonferroni corrections to correct for multiple comparisons (*P* = 0.05 ÷ *k*, where *k* equals the number of comparisons).

## RESULTS

### Participant characteristics

Our study sample was restricted to participants with no missing values for sTfR, CRP, AGP, or malaria (in countries that measured malaria); this resulted in a total loss of 60.0% (11,913 of 29,766) of the observations that met the BRINDA inclusion criteria in PSC and 56.6% (11, 173 of 25,731) in WRA. Subsequently, in an analysis that was restricted to countries that measured AGP, the total sample was further reduced to 9281 in the PSC and 5004 in the WRA because of a lack of AGP data in Mexico and the United States. The pooled sample-size loss was high because the BRINDA inclusion criteria were based on having a measure of one biomarker of iron or vitamin A status; several surveys measured ferritin but not sTfR, which resulted in a loss of 13,935 PSC and 14,402 WRA observations. Participants who were excluded because of missing sTfR, CRP, AGP, or malaria observations did not differ from those who were included with regard to sex or socioeconomic status (data not shown), although children were older (mean age in months ± SD) in the full data set (29.9 ± 15.7 compared with 24.8 ± 14.9 mo, respectively). In PSC, the mean age ranged from 8.3 to 41.5 mo, whereas all WRA were between 15 and 49 y of age with combined mean ages that ranged from 27.2 to 33.5 y (**Table 1**); in PSC, there was considerable age-range variability, notably for the data sets of Bangladesh (6–11 mo), Liberia, Kenya 2007 and 2010 (6–35 mo), and the Philippines (6–23 mo). The proportion of individuals who were classified as having elevated APPs was higher in children than in women. Specifically, 21.2–64.5% of PSC had elevated AGP concentrations, and 5.9–40.4% of PSC had elevated CRP concentrations; 7.2–26.9% of WRA had elevated AGP concentrations, and 7.9–25.7% of WRA had elevated CRP concentrations (Table 1). PSC had a higher prevalence of inflammation on the basis of AGP compared with CRP in all surveys, whereas no clear trend was observed in WRA. In countries that included malaria reporting, the prevalence of malaria in PSC ranged from 19.7% to 32.5% and, for WRA, ranged from 5.0% to 17.9% (Table 1).

**TABLE 1:** Age, inflammation, and malaria in preschool children and women of reproductive age: the BRINDA project^1^

Survey	*n*	Age^2^	CRP concentration >5 mg/L, %	AGP concentration >1 g/L, %	CRP concentration >5 mg/L or AGP concentration >1 g/L, %	Malaria, %
Preschool children						
Bangladesh	1493	8.3 (6–11)	14.3 (11.8, 16.7)^3^	33.4 (29.9, 36.9)	35.8 (32.2, 39.5)	—
Cameroon	774	30.8 (12–59)	37.5 (32.7, 42.3)	39.3 (33.7, 45.0)	48.3 (43.1, 53.5)	25.9 (20.2, 31.5)
Côte d’Ivoire	733	31.7 (6–59)	40.4 (36.5, 44.3)	64.5 (60.3, 68.6)	67.5 (63.8, 71.3)	27.2 (22.3, 32.0)
Kenya 2007	888	19.9 (6–35)	27.8 (23.9, 31.7)	64.2 (60.2, 68.2)	66.0 (61.9, 70.1)	19.7 (15.8, 23.6)
Kenya 2010	843	21.4 (6–35)	34.2 (29.6, 38.7)	60.7 (56.0, 65.4)	61.9 (57.2, 66.6)	32.5 (28.4, 36.6)
Laos	481	33.1 (6–59)	16.6 (11.2, 22.1)	41.7 (34.0, 49.4)	44.0 (36.6, 51.5)	—
Liberia	1434	19.9 (6–35)	29.5 (26.5, 32.5)	56.2 (52.5, 60.0)	59.1 (55.6, 62.7)	29.4 (26.2, 32.6)
Mexico 2006	1588	41.5 (12.7–60)	11.2 (9.0, 13.4)	—	—	—
Papua New Guinea	868	31.4 (6–59)	31.8 (27.4, 36.2)	54.4 (49.6, 59.1)	57.2 (52.8, 61.7)	—
Philippines	1767	15.0 (6–23)	13.9 (11.6, 16.2)	21.2 (17.7, 24.6)	26.0 (22.4, 29.5)	—
United States^4^	1044	38.8 (12–59)	5.9 (4.1, 7.6)	—	—	—
Women of reproductive age						
Cameroon	751	27.2 (15–49)	17.8 (14.8, 20.7)	7.2 (5.1, 9.3)	19.7 (16.6, 22.9)	15.0 (11.3, 18.6)
Côte d’Ivoire	816	27.6 (15–49)	19.7 (16.5, 22.8)	26.9 (23.5, 30.4)	33.7 (29.6, 37.9)	5.0 (3.4, 6.5)
Laos	816	29.3 (15–49)	7.9 (5.6, 10.2)	9.3 (7.1, 11.6)	13.9 (10.9, 16.8)	—
Liberia	1875	28.6 (15–49)	14.3 (12.1, 16.4)	10.4 (8.7, 12.2)	18.5 (16.2, 20.8)	17.9 (15.3, 20.4)
Mexico 2006	3021	31.3 (15–49)	24.2 (21.7, 26.7)	—	—	—
Papua New Guinea	746	29.1 (15–49)	10.0 (7.5, 12.5)	21.9 (18.1, 25.7)	25.0 (21.1, 28.8)	—
United States^4^	3148	33.5 (15–49)	25.7 (23.6, 27.8)	—	—	—

1 AGP, α-1-acid-glycoprotein; BRINDA, Biomarkers Reflecting Inflammation and Nutritional Determinants of Anemia; CRP, C-reactive protein.

2 All values are means (minimums to maximums). Age is given in whole months for children and in whole years for women.

3 95% CIs in parentheses (all such values).

4 From 2003 to 2006.

### Relation between sTfR, inflammation, and malaria

The rank correlations between the 2 APPs ln-CRP and ln-AGP ranged from 0.49 to 0.61 in PSC. Overall, concentrations of sTfR were weakly but positively associated with CRP (ranging from greater than −0.01 to 0.20) and with AGP (ranging from 0.08 to 0.28), and the strength of the relation was stronger for AGP than for CRP across surveys in PSC (**Supplemental Table 1**). The relation between biomarkers was weaker in WRA than in PSC (CRP with AGP: 0.26–0.41; sTfR with CRP: 0.04–0.09; sTfR with AGP: 0.12–0.17). In PSC, malaria had a weak relation (as a dichotomous variable) with sTfR ranging from 0.08 to 0.20, with CRP ranging from 0.17 to 0.28, and with AGP ranging from 0.12 to 0.24; in WRA, the relation of malaria with sTfR and the APPs ranged from 0.01 to 0.10 (Supplemental Table 1).

In the meta-analysis, when individuals were stratified by malaria status and by 4 inflammation groups, the geometric mean of sTfR concentrations was consistently higher for the groups with malaria irrespective of the inflammation level (except for WRA in the late convalescent stage) (**Table 2**). There appeared to be no added effect of both elevated AGP and CRP (the early convalescent stage) on sTfR concentrations irrespective of malaria status.

**TABLE 2:** sTfR in preschool children and women of reproductive age according to inflammation stage and malaria status: the BRINDA project^1^

	Malaria-endemic countries^2^			
	Malaria negative		Malaria positive	
Inflammatory stage	*n*	sTfR, mg/L	*n*	sTfR, mg/L
Preschool children	
Reference	1693	8.42 (6.54, 10.83)^3^	201	10.30 (7.19, 14.76)
Incubation	123	7.79 (5.92, 10.24)	37	10.36 (7.28, 14.73)
Early convalescence	669	9.67 (7.61, 12.29)	666	11.46 (8.38, 15.67)
Late convalescence	992	9.90 (7.71, 12.72)	291	12.34 (8.98, 16.96)
Women of reproductive age	
Reference	2401	7.30 (7.05, 7.55)	267	7.68 (7.07, 8.34)
Incubation	230	7.10 (6.64, 7.59)	76	7.37 (6.65, 8.17)
Early convalescence	190	8.10 (7.42, 8.84)	67	8.68 (7.41, 10.17)
Late convalescence	185	8.17 (7.51, 8.88)	26	7.95 (6.90, 9.17)

1 Reference is defined as a CRP concentration ≤5 mg/L and AGP concentration ≤1 g/L; incubation is defined as a CRP concentration >5 mg/L and AGP concentration ≤1 g/L; early convalescence is defined as a CRP concentration >5 mg/L and AGP concentration >1 g/L; and late convalescence is defined as a CRP concentration ≤5 mg/L and AGP concentration >1 g/L. AGP, α-1-acid-glycoprotein; BRINDA, Biomarkers Reflecting Inflammation and Nutritional Determinants of Anemia; CRP, C-reactive protein; sTfR, soluble transferrin receptor.

2 The following data sets contained information on malaria parasitemia: Cameroon, Côte d’Ivoire, Kenya 2007, Kenya 2010, Liberia (preschool children); Cameroon, Côte d’Ivoire, Liberia (women of reproductive age).

3 Geometric mean; 95% CI in parentheses (all such values).

**FIGURE 1:**
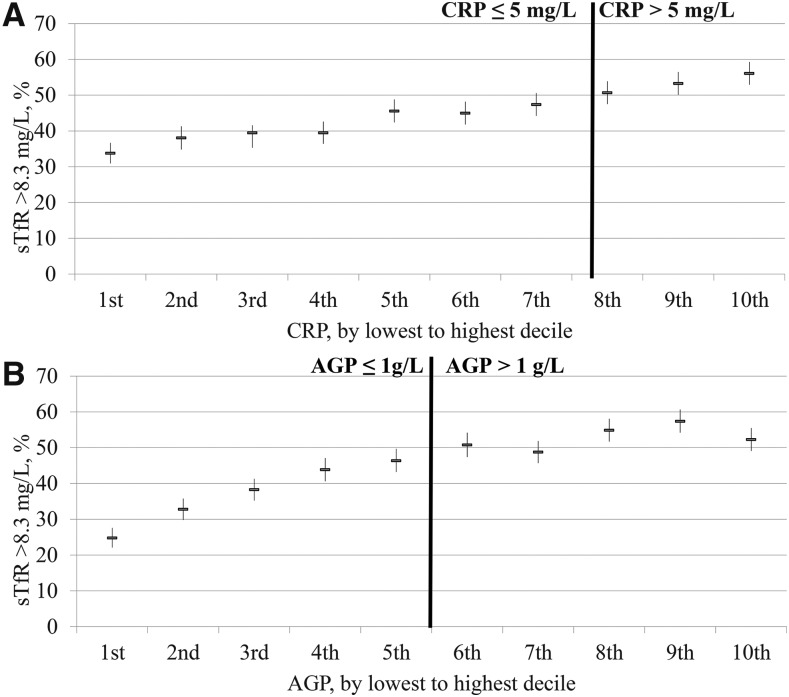
Prevalence of elevated sTfR concentrations in preschool children by CRP (A) and AGP (B) deciles: BRINDA project. Analysis was restricted to surveys (Bangladesh, Cameroon, Côte d’Ivoire, Kenya 2007, Kenya 2010, Laos, Liberia, Philippines, and Papua New Guinea) that measured both CRP and AGP for comparability between CRP and AGP relations with biomarkers (*n* = 9326). AGP, α-1-acid-glycoprotein; BRINDA, Biomarkers Reflecting Inflammation and Nutrition Determinants of Anemia; CRP, C-reactive protein; sTfR, soluble transferrin receptor.

**FIGURE 2:**
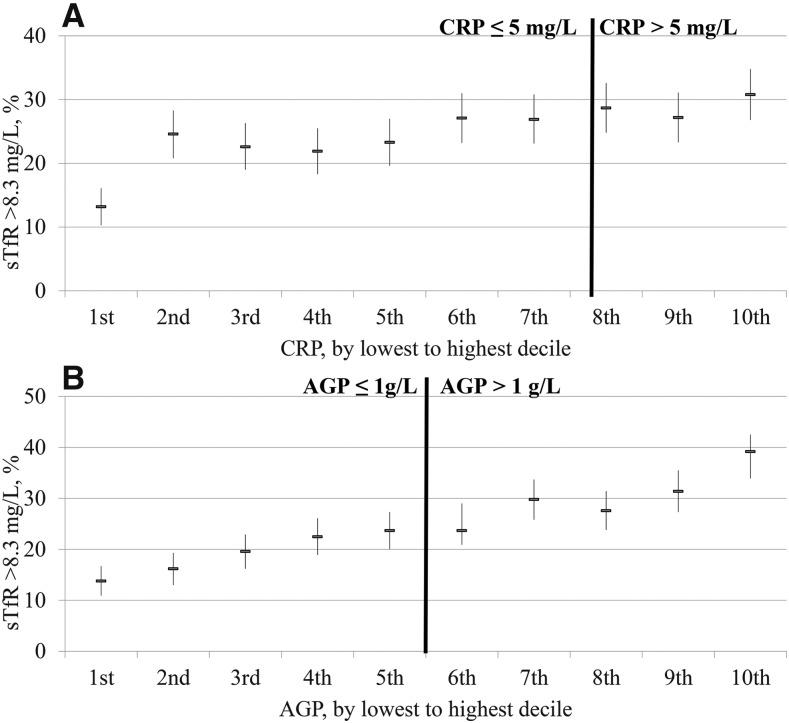
Prevalence of elevated sTfR concentrations in women of reproductive age by CRP (A) and AGP (B) deciles: BRINDA project. Analysis was restricted to surveys [Cameroon, Côte d’Ivoire, Laos (except for retinol-binding protein), Liberia, and Papua New Guinea (except for ferritin)] that measured both CRP and AGP for comparability between CRP and AGP relations with biomarkers (*n* = 5098). AGP, α-1-acid-glycoprotein; BRINDA, Biomarkers Reflecting Inflammation and Nutrition Determinants of Anemia; CRP, C-reactive protein; sTfR, soluble transferrin receptor.

The relation between the estimated prevalence of elevated sTfR concentrations and inflammation deciles was close to linear in PSC (**Figure 1**) and in WRA (**Figure 2**) with the estimated prevalence of iron-deficient erythropoiesis continuously decreasing as the concentrations of CRP and AGP decreased. In the lowest–CRP decile group, the proportion of estimated elevated sTfR concentrations (>8.3 mg/L) was lower (30.8%) than in the highest–CRP decile group (56.1%) in PSC; similarly, the estimated prevalence of iron-deficient erythropoiesis was markedly lower in the lowest AGP decile (24.8%) than in the highest AGP decile (52.3%). The prevalence between highest and lowest decile of inflammation for WRA followed the same pattern as for PSC, albeit less marked; the estimated prevalence of iron-deficient erythropoiesis (sTfR concentration >8.3 mg/L) was 13.2% and 30.8% in the lowest– and highest–CRP-decile groups and 13.8% and 39.2% in the lowest– and highest–AGP decile groups, respectively. The prevalence of an estimated elevated sTfR concentration changed across deciles by a greater percentage with the use of AGP than with the use of CRP (Figures 1 and 2); although there was a weak but clear linear relation between CRP and sTfR, we chose not to adjust sTfR concentrations in the presence of elevated CRP.

### CFs and regression slopes used to adjust sTfR for AGP and malaria

The CFs generated for the ICF-AGP approach, ICF-malaria approach, and ICF-AGP plus malaria approach were <1 across all levels and surveys in PSC and WRA. The regression slopes that were generated for univariate linear regression of ln sTfR resulted in an unstandardized ln-AGP slope from 0.104 (Philippines) to 0.550 (Papua New Guinea) in PSC (**Supplemental Table 2**). Univariate linear regression of ln sTfR resulted in an unstandardized ln-AGP slope from 0.211 (Liberia) to 0.541 (Papua New Guinea) in WRA (Supplemental Table 2). A multivariate analysis of ln sTfR with both ln AGP and malaria in the model resulted in an unstandardized ln-AGP slope from 0.072 (Kenya 2007) to 0.400 (Kenya 2010) and a malaria slope from 0.039 (Côte d’Ivoire) to 0.327 (Cameroon) in PSC (Supplemental Table 2). The addition of malaria to the model dampened the ln-AGP slope in PSC but not in WRA (Supplemental Table 2).

For the BCF, the CF for countries that were not malaria endemic (or where malaria was not assessed) for the group with elevated AGP was 0.86 for PSC and 0.85 for WRA. For malaria-endemic countries where malaria was measured, the CFs were as follows—for PSC: 0.87 (elevated AGP and malaria negative), 0.81 (normal AGP and malaria positive), and 0.71 (elevated AGP and malaria positive); for WRA: 0.89, 0.96, and 0.86, respectively.

### Estimated prevalence of iron-deficient erythropoiesis with the use of AGP to adjust sTfR for inflammation by adjustment approach

**FIGURE 3:**
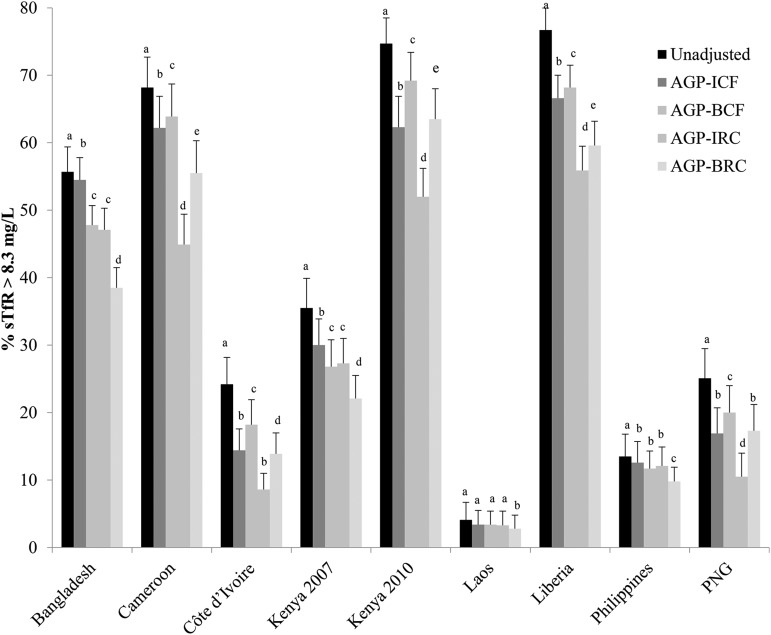
Estimated prevalence [% (95% CI)] of elevated sTfR concentrations with the use of different adjustment factors on the basis of elevated AGP in preschool children: BRINDA project. Surveys are ordered alphabetically. Bars with different lowercase letters within a given country indicate significant differences, *P* < 0.05 (adjusted using Bonferroni correction). AGP-BCF: 0.86 (95% CI: 0.80, 0.93), QE(df = 16) = 2012.3; *P* < 0.0001. Reference value for AGP: −0.52 ln(g/L), QE(df = 10) = 584.5546, *P* < 0.0001. BRC coefficient: ln AGP = 0.28, QE(df = 16) = 2213.5, *P* <0.0001. AGP, α-1-acid-glycoprotein; BCF, Biomarkers Reflecting Inflammation and Nutritional Determinants of Anemia correction factor; BRC, Biomarkers Reflecting Inflammation and Nutritional Determinants of Anemia regression correction; BRINDA, Biomarkers Reflecting Inflammation and Nutrition Determinants of Anemia; ICF, internal correction factor; IRC, internal regression correction; PNG, Papua New Guinea; sTfR, soluble transferrin receptor.

**FIGURE 4:**
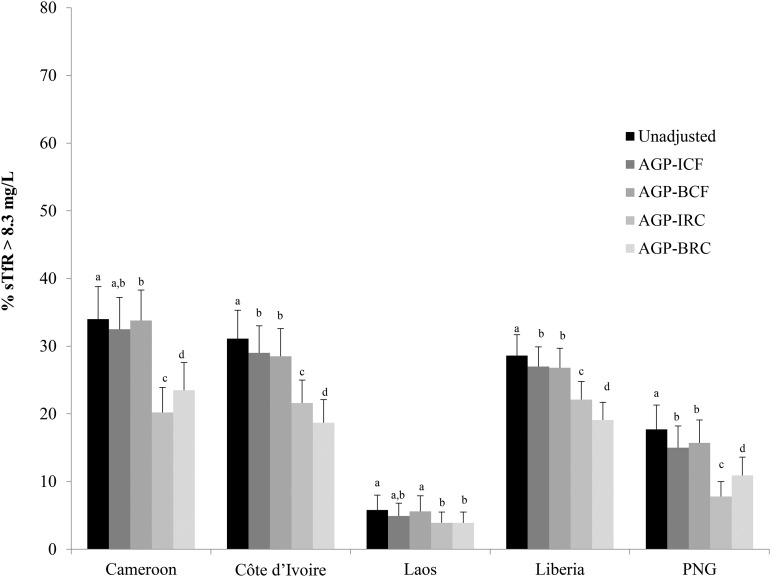
Estimated prevalence [% (95% CI)] of elevated sTfR concentrations with the use of different adjustment factors on the basis of elevated AGP in women of reproductive age: BRINDA project. Surveys are ordered alphabetically. Bars with different lowercase letters within a given country indicate significant differences, *P* < 0.05 (adjusted using Bonferroni correction). AGP-BCF was 0.85 (95% CI: 0.78, 0.92), QE(df = 12) = 945.6350, *P* < 0.0001. Reference value for AGP: −0.63 ln(g/L), QE(df = 4) = 83.78, *P* < 0.0001. BRC coefficient: ln-AGP = 0.33, QE(df = 8) = 692.4, *P* < 0.0001. AGP, α-1-acid-glycoprotein; BCF, Biomarkers Reflecting Inflammation and Nutritional Determinants of Anemia correction factor; BRC, Biomarkers Reflecting Inflammation and Nutritional Determinants of Anemia regression correction; BRINDA, Biomarkers Reflecting Inflammation and Nutrition Determinants of Anemia; ICF, internal correction factor; IRC, internal regression correction; PNG, Papua New Guinea; sTfR, soluble transferrin receptor.

There was a wide variation in the prevalence of unadjusted sTfR concentration >8.3 mg/L, and the prevalence of iron-deficient erythropoiesis was higher in children than in women (in PSC, ranging from 4.1% to 76.7%; and in WRA, ranging from 5.8% to 34.0%) (**Figures 3** and **4**, respectively).

The exclusion of PSC with elevated AGP concentrations (>1 g/L) resulted in a reduction between 21.2% and 64.5% of the sample size, and a smaller loss of data was seen in WRA (7.2–26.9%). Results that excluded individuals with elevated AGP are presented in **Table 3**, and the prevalence estimates were somewhat comparable with those obtained with the ICF-AGP approach as would be expected because the ICF-AGP approach treats AGP as a categorical variable rather than continuous variable.

**TABLE 3:** Estimated prevalence of elevated soluble transferrin receptor unadjusted and after the exclusion of individuals with inflammation in preschool children and women of reproductive age: the BRINDA project^1^

Survey	*n*	Unadjusted, %	Subjects with AGP concentrations >1 g/L, *n* (%)	AGP concentration ≤1 g/L,^2^ %
Preschool children	
Bangladesh	1493	55.7 (52.0, 59.4)^3^	994 (33.4)	53.5 (49.7, 57.4)
Cameroon	774	68.2 (63.7, 72.7)	483 (39.3)	62.3 (57.0, 67.6)
Côte d’Ivoire	733	24.2 (20.2, 28.2)	250 (64.5)	10.0 (5.8, 14.3)
Kenya 2007	888	35.5 (31.1, 39.9)	318 (64.2)	26.1 (20.8, 31.4)
Kenya 2010	843	74.7 (71.0, 78.5)	331 (60.7)	62.8 (56.9, 68.8)
Laos	481	4.1 (1.9, 6.2)	287 (41.7)	3.7 (1.2, 6.2)
Liberia	1434	76.7 (73.5, 80.0)	672 (56.2)	70.2 (65.5, 75.0)
Philippines	1767	13.5 (10.2, 16.8)	1345 (54.4)	11.9 (8.8, 14.9)
Papua New Guinea	868	25.1 (20.7, 29.5)	392 (21.2)	14.3 (10.1, 18.6)
Women of reproductive age	
Cameroon	751	34.0 (29.2, 38.8)	698 (7.2)	32.1 (27.3, 36.9)
Côte d’Ivoire	816	31.1 (27.0, 35.3)	599 (26.9)	28.7 (24.0, 33.3)
Laos	816	5.8 (3.5, 8.0)	734 (9.3)	4.9 (2.8, 7.0)
Liberia	1875	28.6 (25.4, 31.7)	1677 (10.4)	27.0 (23.9, 30.1)
Papua New Guinea	746	17.7 (14.2, 21.3)	584 (21.9)	15.2 (11.9, 18.4)

1 AGP, α-1-acid-glycoprotein; BRINDA, Biomarkers Reflecting Inflammation and Nutritional Determinants of Anemia.

2 Elevated soluble transferrin receptor concentrations (>8.3 mg/L) in the subset of the sample with nonelevated inflammation.

3 95% CIs in parentheses (all such values).

The estimated prevalence of an elevated sTfR concentration (>8.3 mg/L) consistently decreased, albeit not always significantly, in PSC when adjusted for elevated AGP concentrations compared with unadjusted concentrations in all surveys regardless of the adjustment method (Figure 3).

In PSC, the application of the ICF-AGP approach to adjust the sTfR concentrations decreased the prevalence of unadjusted compared with adjusted iron-deficient erythropoiesis estimates by an absolute median decrease of 6.0 percentage points (pps) (range: 0.7–12.4 pps). Unadjusted to adjusted prevalence estimates changed by as little as 1 absolute pp in 3 surveys (55.7–54.5% in Bangladesh; 4.1–3.4% in Laos, and 13.5–12.6% in the Philippines) to more-substantial decreases such as from 74.7% to 62.3% in Kenya 2010. The decrease in the estimated prevalence of iron-deficient erythropoiesis in WRA was much lower with an absolute median decrease of 1.6 pps (range: 0.9–2.7 pps).

In PSC, the adjustment of sTfR concentrations with the use of the ICF-AGP approach compared with the BCF-AGP approach resulted in a median absolute decrease of 1.6 pps (range: −6.9 to 6.7 pps); there was as little as no change (in Laos), a small decrease (12.6–11.7% in the Philippines), substantial decreases (54.5–47.8% in Bangladesh; 30.0–26.8% Kenya 2007), or increases (other data sets ranging from 1.6% to 6.9%) (Figure 3, **Supplemental Table 3**) in prevalence estimates. The difference in the prevalence estimates between the application of adjustments with the use of ICF-AGP and BCF-AGP approaches was negligible in WRA with an absolute pp difference <1 across all surveys (Supplemental Table 3, Figure 4).

In PSC, when the IRC-AGP approach was used to adjust sTfR concentrations, the prevalence of unadjusted compared with adjusted iron-deficient erythropoiesis estimates decreased considerably in most countries (absolute median difference: 14.6 pps; range: 0.8–22.7 pps) with the greatest change in Kenya 2010 (from 74.7% to 52.0%) and the lowest absolute percentage change in Laos (4.1–3.3%). When applying the BRC-AGP approach, the absolute median decrease was 11.2 pp (range: 1.3–17.2 pp) with the greatest prevalence decrease observed in Liberia (77–60%) and the lowest prevalence decrease in Laos (4–3%) in PSC (Figure 3, **Supplemental Table 4**). When applying the IRC-AGP approach to adjust sTfR concentrations in WRA, the prevalence of iron-deficient erythropoiesis decreased by as absolute median of 9.5 pps from a little as 1.9 pps (5.8–3.9% in Laos) to as much as 13.8 absolute pps (34.0–20.2% in Cameroon) compared with unadjusted prevalences, and similar results were shown with the use of the BRC-AGP approach (absolute median difference: 9.5 pps; range: 1.9–12.4 pps) (Figure 4, Supplemental Table 4). A comparison of differences with the use of the IRC-AGP approach compared with the BRC-AGP approach to adjust sTfR concentrations resulted in an iron-deficient erythropoiesis absolute median decrease of 3.7 pps (range: −11.5 to 8.6 pps) with the BRC-AGP approach in PSC; whereas in WRA, the absolute median decrease was 0 pps (range: −3.3 to 2.9 pps). Finally, the comparison of the absolute median difference between ICF and IRC was 6.4 pps (from 0.1 pps in Laos to 17.3 pps in Cameroon) for PSC and 7.2 pps (from 1 pps in Laos to 12.3 pps in Cameroon) for WRA.

### Estimated prevalence of iron-deficient erythropoiesis with the use of AGP and malaria to adjust sTfR for inflammation by adjustment approach

**FIGURE 5:**
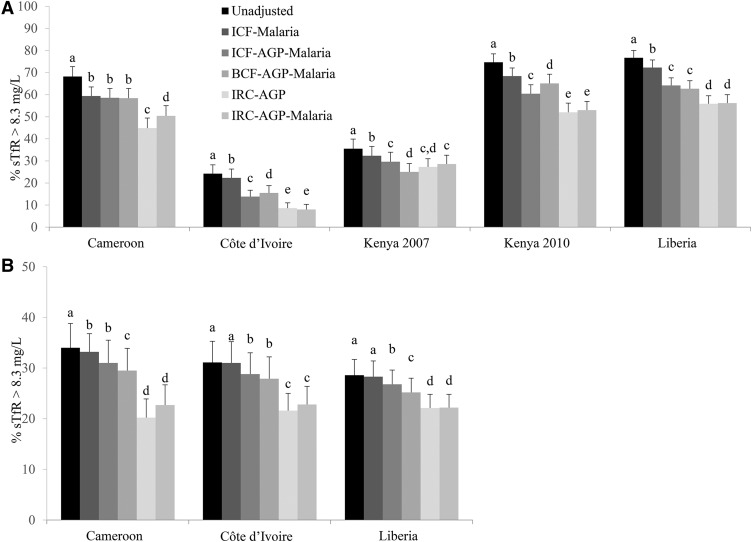
Estimated prevalence (%) of elevated sTfR concentrations with the use of different inflammation and malaria adjustment approaches in preschool children (A) and women of reproductive age (B): BRINDA project. Surveys are ordered alphabetically. Bars with different lowercase letters within a given country indicate significant differences, *P* < 0.05 (Bonferroni correction). AGP, α-1-acid-glycoprotein; BCF, Biomarkers Reflecting Inflammation and Nutritional Determinants of Anemia correction factor; BRINDA, Biomarkers Reflecting Inflammation and Nutrition Determinants of Anemia; ICF, internal correction factor; IRC, internal regression correction; sTfR, soluble transferrin receptor.

The application of a malaria CF to sTfR concentrations in countries where malaria was measured resulted in a median decrease of 4.4 pps with the lowest absolute percentage difference of 1.9 pps in Côte d’Ivoire (24–22%) and the highest absolute percentage difference of 8.8 pps in Cameroon (68–59%) between unadjusted and adjusted iron-deficient erythropoiesis estimates in PSC and an absolute median decrease of 0.3 pps (range: 0.1–1.8 pps) for WRA. When both malaria and AGP were included in the CF model (both ICF and BCF), the absolute prevalence difference between unadjusted and adjusted iron-deficient erythropoiesis was an absolute median decrease of 10.4 pps (range: 5.9–14.3 pps) for PSC and of 2.3 pps (range: 1.8–3.0 pps) for WRA (**Figure 5**, **Supplemental Table 5**).

When we compared sTfR concentrations that were adjusted for malaria and inflammation with IRC approach, the iron-deficient erythropoiesis prevalence in PSC generally decreased more than with either of the CF approaches (ICF or BCF). However, accounting for both malaria and AGP in the IRC approach had a more modest effect on the decrease in prevalence of iron-deficient erythropoiesis than was shown in the IRC approach in which only AGP was accounted for. Adjustment for AGP alone with the use of IRC produced similar point estimates of elevated sTfR as were produced with the use of both AGP and malaria in the RC (Figure 5). The decrease in the prevalence of iron-deficient erythropoiesis after adjustment for malaria and AGP (IRC approach) resulted in a median decrease of 14.2 pps (range: 6.3–18.1 pps) compared with that of unadjusted iron-deficient erythropoiesis in PSC and a corresponding absolute median decrease of 9.5 pps (range: 6.4–14.2 pps) in WRA.

### Estimated prevalence of iron-deficient erythropoiesis with the use of the IRC approach to adjust sTfR for AGP taking into account confounders

In nearly all surveys, there was no change in the prevalence of elevated sTfR adjusted for AGP with the use of the IRC approach when controlling for confounders (socioeconomic status, maternal education, age, and sex when available; data not shown). The absolute median difference of adjusted elevated sTfR while controlling for confounders compared with not controlling for confounders was 0.1 pp (range: −0.4 to 2.0 pps) for the IRC-AGP approach in PSC in all surveys except for in Cameroon. Cameroon PSC showed a median difference of 6.7 pps. The absolute median difference of depleted iron stores compared with the difference when these factors were not controlled was −0.2 pps (range: −0.2 to 0 pps) for the IRC-AGP approach in WRA.

## DISCUSSION

We conducted a multicountry analysis with various approaches to correct sTfR concentrations in the presence of malaria or inflammation (or both) because of the challenges in accurately estimating iron-deficient erythropoiesis with the use of sTfR. Adjustment for inflammation or malaria, regardless of the approach explored, resulted in a reduced prevalence of elevated sTfR concentrations in PSC and WRA, although the reduction was more marked in PSC. These findings were strengthened by the large sample size of the data set compared with in previous studies with similar findings (9–11). sTfR has been considered by many researchers as a potentially useful biomarker in settings where there is a high prevalence of inflammation because (unlike ferritin) it is not an APP and, therefore, is thought to not (28) or inconsistently (29) be influenced by inflammation. Notwithstanding, we showed that sTfR has a weak but consistent relation with AGP concentrations, which is associated with a lower prevalence of iron deficiency on the basis of measurements of sTfR, thereby indicating the need to apply adjustments for inflammation. Note that sTfR is a biomarker of iron-deficient erythropoiesis; thus, the use of sTfR as a proxy for nutritional iron deficiency should be carefully evaluated, especially in the absence of adjustments for other factors that cause erythropoiesis.

In our data, despite a relation between CRP and sTfR, the magnitude of the difference in the prevalence of iron-deficient erythropoiesis, when adjusting concentrations of sTfR with the use of CRP, was minimal and not consistent across surveys. CRP is highly responsive to the rise in cytokines at the start of an acute-phase response, and elevations in both CRP and cytokine concentrations suppress erythropoietin production, thereby preventing the elevation of plasma and serum concentrations of sTfR, which results in a temporary but complete shutdown of normal iron metabolism (30). In addition, the magnitude of the difference in iron-deficient erythropoiesis was minimal when adjusting for CRP, which is a finding that was previously shown (31, 32). Although sTfR is not an APP, it may be affected by inflammation through alternative mechanisms. Kasvosve et al. (33) proposed that an increase in transferrin receptor expression could occur as a result of the redistribution of iron or the upregulation of the hypoxia inducible factor 1-α in response to inflammation. Therefore, to summarize the first 2 research questions that we posed, our findings suggest that concentrations of sTfR should be adjusted in the presence of inflammation and that only AGP, and not CRP, is needed to correct sTfR concentrations.

In response to question *3*, we showed that, although correcting for AGP alone with the use of IRC may be sufficient in malarial areas, additional adjustment for malaria needs further exploration on the basis of biological plausibility. Other studies have evaluated the influence of malaria above and beyond the influence of inflammation on sTfR concentrations. For example, an isotope study that provided labeled iron to anemic children with and without malaria showed a significant reduction in sTfR concentrations at day 1 in the malaria group, thereby indicating reduced erythropoiesis (34). By day 15 of iron supplementation, sTfR concentrations decreased in the nonmalarial group and increased in the malarial group, which were thought to be due to the degree in the drop of erythropoietin concentrations. Beguin (35) postulated that such changes are not directly due to the acute-phase response but indirectly because of reduced erythropoietin production and erythropoiesis suppression as part of the inflammatory cascade. Further, the induction of the iron-regulatory hormone hepcidin causes a decrease in iron absorption and the sequestering of iron in the macrophages and hepatocytes. In addition, the processing of iron in the macrophages is slower in parasitized red cells. In infected cells, iron is in the form of hemoglobin and hemozoin, but the hemozoin cannot be broken down by heme oxygenase (36, 37). These mechanisms prevent iron from being recycled for erythropoiesis. Specific to sTfR, malaria results in enhanced erythropoiesis in response to hemolysis (37, 38), which potentially explains the consistently higher mean sTfR concentrations that have been shown after the initial acute infection and supports the need for the inclusion of malarial data in the adjustment approaches.

To answer our fourth research question, we compared a number of approaches to adjust sTfR concentrations in the presence of inflammation or malaria. Although all methods resulted in lower estimates of the prevalence of iron-deficient erythropoiesis, we could not single out the most valid method without a gold standard. Because of this challenge, other factors may need to be considered when selecting an adjustment method. The feasibility and ability to implement the correction are important criteria; further, the capability of the approach to reflect the severity of inflammation can also be considered. On the basis of these considerations, we suggest that AGP should be included in any sTfR adjustment, and in malaria-endemic countries, the presence or absence of malaria parasites should be taken into account. The regression approach uses a continuous system to adjust for inflammation rather than establishing a categorical system and, as such, incorporates the intensity of inflammation. Because of the heterogeneity of the data across surveys, the survey-specific or internal RC approach is likely to yield the most-valid results. However, this is also the most complicated approach and requires statistical expertise such that this approach may only be suitable in a research context. For stakeholders implementing surveys with less statistical capacity, applying the ICF by creating 2 groups in nonmalarial contexts (normal AGP and elevated AGP) or 4 groups in malaria endemicity (malaria negative and normal AGP, elevated AGP and malaria negative, normal AGP and malaria positive, and elevated AGP and malaria positive) and subsequently calculating the CFs as the ratio of geometric means of the reference group (malaria negative and normal AGP) to those of the respective other groups may be a more feasible option. When there is limited statistical capacity and relatively small sample sizes, the use of CFs calculated in this meta-analysis may be more practical than applying the RC technique. Because of the large heterogeneity of individual slopes between sTfR and AGP across countries, the application of an external slope (BRC approach) is not suggested at this time.

Although there are reports that have suggested the use of a higher sTfR cutoff for young children (39, 40), we did not calculate the prevalence with the use of higher cutoffs. Although the prevalence of iron-deficient erythropoiesis would be reduced with the use of such an approach, the underlying influences of inflammation and malaria will be similar and, as such, are not necessarily helpful in addressing the questions about whether and how sTfR is to be adjusted for inflammation and malaria. Nonetheless, further investigation clarifying the most appropriate sTfR cutoff in younger children should be conducted to either confirm or invalidate the currently used cutoff.

Limitations to our study include the use of cross-sectional data only because no longitudinal data were available that fit the criteria and the absence of a gold standard to compare adjustment algorithms to assess their validity in diagnosing true iron deficiency. In addition, although a large sample size was used for these analyses, more than one-half of the observations from the total BRINDA data set were lost because several surveys only measured ferritin for identifying iron deficiency. Several smaller studies have evaluated the performance of sTfR in diagnosing iron deficiency against bone marrow staining, some of which have been referenced above in the introduction section, but the vast majority of these studies included study participants of limited comparability (e.g., severe anemia only, pregnancy, and HIV-positive subjects only) with a population that has been typically included in population-based surveys; some of the studies looked at measures of inflammation, but again, the limited comparability of the study population renders a generalizable interpretation difficult. There is a need to identify how to best validate any adjustment to inflammation when attempting to diagnose iron deficiency in populations who are exposed to recurrent infections.

In conclusion, despite the limitations inherent in the data available for this work, it is an important contribution to the issue of assessing iron status in the context of subclinical inflammation and malaria. However, it is even more evident that considerably more research is needed to appropriately address emerging questions about iron-status assessment in contexts of a high inflammation burden in different population groups.
